# Psychological Capital of MIS Development Teams, System Effectiveness, and Social Innovation: A Systematic Literature Review

**DOI:** 10.3389/fpsyg.2019.01436

**Published:** 2019-07-12

**Authors:** Yuan Tang, Yun-Fei Shao

**Affiliations:** ^1^School of Management and Economics, University of Electronic Science and Technology of China, Chengdu, China; ^2^Institute of Management, Sichuan University of Science and Engineering, Zigong, China

**Keywords:** psychological capital, information system effectiveness, social innovation success, MIS staffs, inter-organizational management

## Abstract

Inter-organizational information systems are critical in the modern business world, as a large portion of economic activities are done through inter-organizational collaborations. One such important collaboration is social innovation/entrepreneurship, which involves multiple parties from diverse areas cooperating in major tasks. Against such a backdrop, exploring beneficial factors for organizational members to actively develop and/or make good use of a management information system (MIS) for collaborative performance has become a vital research question. This conceptual paper argues that positive psychology is critical to encourage members to volunteer to construct an MIS that facilitates social innovation effectiveness. Specifically, we discuss the four dimensions of psychological capital (i.e., hope, optimism, self-efficacy, and resilience) on effective inter-organizational MIS attributes (e.g., connectivity), and on the consequences of social innovation. At the core of this discussion, we believe that a positive psychological foundation is a driver for staff effort to contribute to a better MIS, which could benefit social innovation success. This research contributes by offering inspiration for future studies to link the micro- and macro-level aspects of social innovation and entrepreneurship phenomena.

## Introduction

### Significance of Inter-Organizational Management Information System (MIS) in Social Innovation

In a highly competitive business world, enterprises strive to establish practices such as producing commodities tailored to meet the social needs of customers (Prajogo et al., 2018). It should be appreciated that any business has a prime objective of satisfying its customers. This means that an inter-organizational information system is useful for ensuring that modern corporations meet this vital objective. With the goal of producing goods that are intended to address social needs, companies have a better chance to establish a niche in such needs when they interact with and/or exchange ideas through a jointly run management information system (MIS).

In order to come up with a product that meets the social needs of consumers, a technology that is non-existent in the organization might be needed. Inter-organizational management information systems employ technology and intelligent computer systems to convey information in and around the operations of partnered organizations. It follows, therefore, that technological advancement in one company can easily be accessed by another company that essentially lacks such technology. Additionally, such information is conveyed quickly, since companies are able to access it from a shared information system without prompt (Prajogo et al., 2018).

Over the past few years, the scientific community (and recent policy makers) have become increasingly interested in the concept of social innovation (Borzaga and Bodini, 2014). Often, a company’s survival is explained by the “improvement of innovation” that pursues profit and competitive advantage. In contrast, social innovation is triggered by an interest in improving social well-being (Dawson and Daniel, 2010). Unlike profit-driven or competitive business-driven innovation, social innovation is often triggered by concerns about people and communities rather than commercial interests. An adopted or developed social innovation, such as online procurement, will only be desirable to an organization’s customers if trade around it is efficient (Prajogo et al., 2018). This efficiency can be brought forth through the use of Inter-organizational MIS. Organizations that depend on each other can network and share information amongst themselves in a bid to ensure that information transmission is automated. It follows, therefore, that distribution of socially innovated products will require minimal manual input. Standardized and automated procedures of production and distribution ensure that goods produced are of high quality, while at the same time ensure there is timely and efficient delivery.

Inter-organizational MIS ensures that there is healthy and sustainable competition in as far as production of goods intended to meet social needs is concerned (Zhang et al., 2016; Vanpoucke et al., 2017; Okwir et al., 2018). Tentatively, partnered businesses are able to pool their resources together to promote the sales of their socially innovated products by mounting a joint effort in offsetting competition from rivals (Rui and Yip, 2008). This can be done through jointly sponsored online product marketing campaigns funded by partnered businesses.

Finally, a successful social innovation effort requires a rich pool of information that can only be accessed if there is a connection to global information (Luthans and Youssef, 2004; Zhang et al., 2016). Tentatively, MIS makes information available on a global scale. This means that regardless of the level of commencement of the MIS communication level, it can gradually be transformed into a global information network by, for instance, hiring more staff and buying advanced technological tools, such as satellites.

In sum, organizations need to build inter-organizational IS for successful social innovation, especially for long-term social innovation projects, and to implement efficient and effective coordination. The EDI or SCM were both such kind of inter-organizational systems. Beyond those, examples of inter-organizational IS include inter-organizational knowledge management system, collective decision support systems, and others. Without those inter-organizational IS of different kinds and purposes, the multiple parties engaging in a social innovation project could not coordinate well.

### Significance of Exploring Successful Factors for Inter-Organizational MIS for Social Innovation Success

There are several reasons why exploring areas where inter-organizational MIS has had a positive impact is recommended for social innovation success (Iqbal et al., 2015). First, institutional tensions are prevalent in many organizations as well as partnerships. Such tensions may act as a stumbling block to the institutionalization of any social innovative campaigns. It is, therefore, prudent that sources of institutional tension that are related to social innovations be pinpointed and, most importantly, be dealt with. That said, it is important to be cautiously aware of failed MIS systems when spearheading social innovation.

Human beings are naturally motivated by good (Stadtler, 2014). In essence, providing successful cases of use of MIS in institutionalizing social innovation will go a long way in convincing organizational stakeholders that social innovative campaigns and projects are achievable when the right approaches are adopted (Yang and Maxwell, 2011). The social impact of innovation and change is inevitably a complex process, and the goal of actively integrating social benefits into the development and application of new products and services poses varying degrees of challenge to the business agenda (Dawson and Daniel, 2010). It follows, therefore, that the confidence of stakeholders in the process will be bolstered, and as such, they will be in a position to accord their support to the MIS project for social change.

Successful factors for inter-organizational MIS for social innovation rely on organizational culture and ethics put in place in executing such MIS campaigns for social innovation (Gil-Garcia and Sayogo, 2016). It follows, therefore, that through exploration of these success factors, members of the organization will be sensitized in advance to the expected professional standards and codes of ethics in adopting an inter-organizational MIS campaign for social innovation.

Lastly, not every inter-organizational MIS will yield the desired impact on the social innovation objectives of a business. This means that adopting the wrong approach can be detrimental to the long term sustainability of partnered businesses. The capital outlay of venturing into a joint MIS project goes to waste in the event that the campaign is not meeting its short term or long term objectives (Gil-Garcia and Sayogo, 2016).

### Significance of the Psychological Capital Perspective in the Study

The states of mind of individuals in the group tasked with the responsibility of developing the joint MIS should be positive (Goldsmith et al., 1998). The author is of the opinion that this will enable the group to accord the task in hand with unparalleled attention. The first and most important reason for considering psychological capital is that it goes a long way in guaranteeing success, in that the team of individuals tasked with the MIS development must be willing and able to do so (Zhang et al., 2016).

Secondly, organizational behavior, such as organizational citizenship and altruism, are both important in ensuring the success of the MIS campaign (Goldsmith et al., 1998; Haider et al., 2017; Hwang and Choi, 2017). According to Goldsmith et al. (1998), the MIS development team will not only develop the program but also be at the forefront of its implementation if they are loyal to the organization. Civic virtue, on the other hand, ensures that the MIS development team is resilient to challenges and is optimistic that the effort put in place will yield positive social innovative results in the long run. According to Goldsmith et al. (1998), psychological capital examination helps in establishing individuals with extra role behavior. Such individuals are able to carry out the tasks of developing the MIS without supervision, while at the same time demonstrating a willingness to go the extra mile to foster success.

**FIGURE 1:**
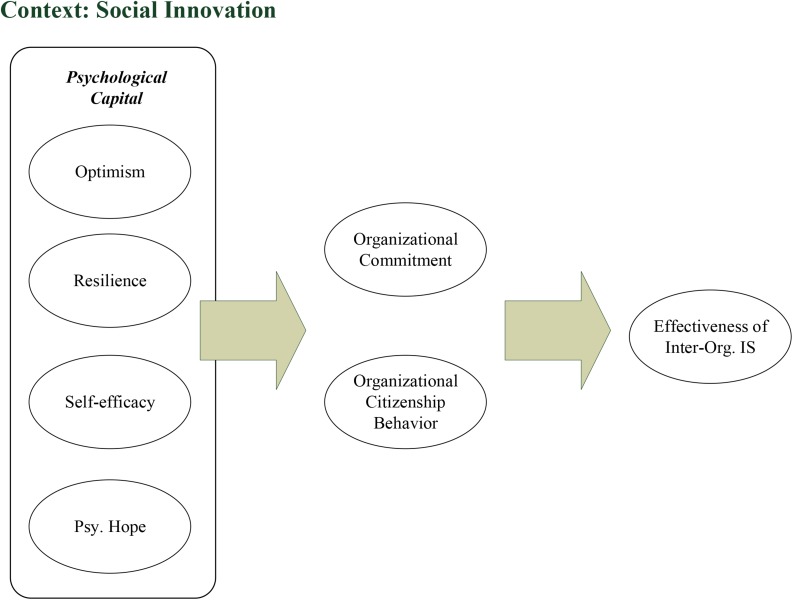
Research Concept. Inter-org IS refers to inter-organizational information system.

The success of an MIS in fostering social innovation is dependent on the development team to find within them a renewed interest in the value of being positive (Goldsmith et al., 1998; Seidler-de Alwis and Hartmann, 2008; Chen, 2016). What follows is that they will find a profound strength and an improvement in psychological capabilities that will further place them in a better position to develop the proposed inter-organizational IS. The research concept of this research is shown in Figure 1.

## Literature Review

According to Fitzenz (2000), human capital is comprised of four major subdivisions, namely psychological capital, intellectual capital, emotional capital, and social capital, which together are simply referred to as “PIES.” In order to resolve various human problems in a given association, the theory of psychological capital is projected as an essential subdivision that can be applied to resolve such issues. Additionally, psychological capital can also be referred to as a person’s optimistic psychological state of improvement that includes various measurements. These measurements mainly include self-efficacy or confidence, hope, resiliency, and optimism (Liu et al., 2009; Newman et al., 2014). Optimistic psychology programs convey increased awareness concerning the importance that they have on human functioning. The four psychological capital dimensions are explained in the following sections.

### Confidence/Self-Efficacy

Bandura (1997) defines confidence or self-efficacy as the belief that various individuals have when they implement some actions and a positive outcome is attained. Confident individuals usually opt to select difficult tasks in order to achieve their intentions. Self-reliant people always withstand difficulties and make sure their goals and objectives are accomplished. Moreover, Bandura (2007) discovered that self-confidence contributes largely to human performance factors, which include aspirational goals and anticipated opportunities in a particular venture. Through their meta-analysis comprising 114 studies and 21,616 subjects, Stajkovic and Luthans (1998) noted that there was a weighted average correlation of approximately 0.38 between confidence and human beings’ performance results.

### Hope

Hope is a combination of both determination and will power. According to Ybasco et al. (1996), hope is comprised of both human beings’ willpower to ensure that their goals are attained and the desire they have to come up with alternative ideas to achieve their goals. Hope is an inspirational state that consists of three main features: agency, paths, and objectives. Normally, hopeful individuals have the agency or wish to accomplish their objectives and are capable of formulating specific pathways or approaches toward achieving their goals.

### Optimism

Optimism is defined by Luthans and Youssef (2004) as a form of a descriptive style that is strictly associated with affirmative occurrences to interior, permanent, and persistent causes and negative occurrences to exterior, temporary, and specified situations. Optimistic individuals take tribute for the good things that occur in their lives, so that their self-confidence is enhanced, and at the same time they detach themselves from bad things that occur, especially toward new technology adoption and usage (Chen et al., 2009, 2013). Optimism is therefore concomitant with an individual’s performance, as discussed by Martin-Krumm et al. (2003) and Schaupp and Carter (2010).

### Resilience

Resilience is referred to as the capability individuals have to overcome all their misfortune, their failure, and in most cases the ability to start taking responsibility by opting to change their lives. Most resilient people have a strong belief that despite all the challenges that they face in life, there is always a pathway that can be implemented in order to overcome them (Luthans, 2002; Luthans et al., 2007; Peterson et al., 2008). Various administrative intellectuals in a recent research analysis have concluded that resilient persons are able to overcome all the challenges brought their way.

In the above psychological capital (PsyCap) elements, there is a clear relationship between psychological strengths and the positive results realized in various working areas, as noted by Peterson et al. (2008). For instance, according to Peterson et al. (2008), most politicians’ or societal leaders’ hope is related to how businesses perform in the financial sector, worker contentment, and worker resiliency in the business. Additionally, in a Chinese study about manufacturing workers, Luthans and Youssef (2004) noted that there was a significant relationship between employee ranks of resiliency and the work performance in organizations. On the other hand, Seligman (2011) found a clear link between the optimism measured and the worker’s performance, specifically in insurance organizations.

From the above scholars who carried out the research, it is appropriate to conclude that Psychological capital has an impact on preventing employee non-attendance at their places of work and their desire to quit their jobs (Hu et al., 2018; Bockorny and Youssef-Morgan, 2019; Carmona-Halty et al., 2019). Moreover, from the research on PsyCap elements, it evident that PsyCap is positively associated with issues of job commitment, satisfaction, behaviors of individuals in an organization, leadership efficiency, and the performance of workers in their organizations (Luthans and Youssef, 2004; Luthans et al., 2006; Peterson et al., 2008). These outcomes of PsyCap elements are similar to results from research carried out in China (Lifeng, 2007), although, analytical results about India were not found. It is also clear that all the psychological capital elements are states, and not temperaments, according to Luthans (2002); hence, these elements might be influenced by different organizational contexts, for instance, in both the private and the public sectors.

### Organizational Commitment

Organization commitment is where employees tend to strongly attach themselves in various organizations through the satisfaction that they get from their jobs (O’Reilly and Chatman, 1986; McDonald and Makin, 2000; Saks, 2006; Bulut and Culha, 2010). Allen and John (1997) formulated three specific component models concerning the organizational commitment of employees. These models included effective, continuous, and normative commitment. Effective commitment in general entails the linkage that the workers have with their organizations and what they feel about the organization as a result of their personal desires. Continuous commitment is a situation where individuals working in an organization are mainly attached to it due to the favors the organization offers to them in relation to the challenges they undergo when they attempt to quit. Lastly, the normative commitment is a moral ideology that employees have, where they remain in a given organization not because they are comfortable or benefit from it, but because they think remaining there is the best and right thing to do (Islam et al., 2014).

According to Luthans (2002), organizational commitment has been found to correlate with all the psychological capital elements, such as self-efficacy or confidence, hope, optimism, and resilience. After conducting research on approximately 167 managers in various organizations, Sinha et al. (2002) noted that there was a specific relationship between self-confidence and the organizational commitment. There was insufficient evidence in the study concerning the effect of the affective, normative, and continuous commitment on the Psychological capital elements in India. Additionally, conclusions can be made following the above research on the psychological capital elements that, despite the difficulties and setbacks, have implications that are different in both the private and public organization commitment context (Lyons et al., 2006). It is noted that the implications of psychological capital on the organizational commitment vary in different organizations.

Generally, MIS from different organizations would not work with a similar organizational commitment. Thus, a collectively positive psychological capital as a shared base for cognitive and affective common ground toward social innovation issues becomes critical for inter-organizational MIS staffs’ commitment building. This argument is among the core points that we proposed in this article. With psychological capital that could function across organizational boundaries, commitment toward the whole collaborated social innovation project could be formed among people staffs from different organizations.

### Organizational Citizenship Behavior

Organizational citizenship behavior is an important aspect in a given organization, and it was first observed in the 1980s. Organizational citizenship behavior is where the behaviors of employees in a workplace affect the organization, either positively or negatively, depending on whether the behaviors are desirable or not (Ensher et al., 2001; Lee and Allen, 2002; Spector and Fox, 2002; Avey et al., 2008, 2011; Podsakoff et al., 2009; Bolino et al., 2013). These behaviors consist of assisting fellow workers to complete a given project on time through teamwork, advising fellow employees on important issues when it is certain they are wrong, and also ensuring given tasks are performed with positive outcomes (Organ and Ryan, 1995). After conducting research on approximately 132 employees, Avey et al. (2008) concluded that psychological capital was directly related to arrogance and employee behavior in various organizations. Bowling et al. (2012) also concluded that organizational citizenship behavior was correlated with self-confidence of employees in each organization. From the research on organizational citizenship behavior, it can be concluded that behaviors differ in both public and private organizations. For a given organization to be successful, it is of utmost importance for various stakeholders in the company, such as the employees, to practice positive behaviors, and work as a team in order to realize the goals of their organization.

Several researchers carried out investigations concerning the effect that the psychological capital measurements had on both private and public organizations. The researchers used different tools during their investigation. Ybasco et al. (1996) measured hope using a hope scale, and had approximately six items comprised of an equal number of pathways and agencies. Neill and Dias (2001) used the scale to measure the resilience tool, which had 15 items. Self-confidence was also measured using the Jerusalem and Schwarzer (1992) confidence scale and had 10 items. Optimism was also measured by Scheier and Carver (Scheier et al., 1995) and had a total of eight items. Each organizational commitment was measured with a scale comprised of 18 items largely based on the three measurements of organization commitment – normative, continuous, and affective commitment – that had an equal number of six items. With the help of Chattopadhyay (1999), another important factor that was measured was organizational citizenship behavior. This factor had approximately 35 items based mainly on five measurements, which included Self-sacrifice (11 items), justice (five items), self-esteem (six items), peer relations (six items), and interdependence (six items).

After the research was conducted on the two organizations, it was evident that apart from resiliency, all the other measurements, including confidence, hope, and optimism, were dissimilar in both the public and the private sector. From this statement, we can see that there is still a similarity between our first propositions by Luthans and Youssef (2004), who that noted that psychological capital measurements are states, not trait temperaments. We therefore conclude that different organizations have different levels of effort made or applied to ensure they develop psychological capital.

Considering the self-confidence issue, Bandura (2007) noted that confidence could be freely implemented in various organizations. The researcher continually argued that for confidence to develop in organizations, it is necessary for workers to learn more in order to achieve the goals of a given organization. The issue of self-confidence was also the same in the results obtained by researchers from various organizations. According to those results, it was evident that worker self-confidence was greater in public organizations than in the private sector.

In terms of hope in psychological capital, the research made it clear that hope could also be developed in different organizations depending on how strictly the approaches to achieve this were planned (Luthans et al., 2006). It was evident that PsyCap hope was greater in the private sector compared to the public sector. This was due to the several reports that workers in the private sector took part in the process of setting organizational goals.

Based on the literature review, we develop propositions based on the following conceptual scheme.

## Proposition Development

### Optimism Positively Facilitates Inter-Organization MIS for Social Innovation Success

Optimism is a key Psychological capital element; it is the ability of an individual to be able to perceive the best outcome in every situation that occurs (Luthans and Youssef, 2004). It is a significant factor that facilitates work performance to enhance social innovation success (Martin-Krumm et al., 2003). Various optimistic people are always productive in organizations, since they are able to venture into difficult tasks and risky options in business, which lead to positive end results.

Optimism is an important factor in the workplace, since it is able to create an environment that promotes creativity and innovation (Gupta and Singh, 2014). Being optimistic is a factor that leads to trusting a person’s talent and beliefs to achieve outcomes in a specific area. It is through this trust and belief that workers are encouraged to work and to improve in the work force. This encourages them to seek better intuitive ideas benefitting the workforce, since by doing so, the goal of social innovation is achieved.

Through the practice of optimism in the office space, an atmosphere of trust is created in the workplace, and such a condition is favorable to the employers and workers. For example, in the situation whereby leeway to work freely within an organization is offered by the employers to the workers, employees are able to work freely and more effectively, since they know they have the trust of the employers.

Optimism has led to the success of social innovation, since it helps employees be more resilient in given organizations. For example, when faced with challenges at work, they are able to look at the positive sides enabling them to overcome all the setbacks in their way. This enhances performance in the organization, leading to improved social connections among employees.

Moreover, Optimism has a positive link to organizational citizen behaviors (Jung and Yoon, 2015). Optimistic people are able to offer good advice or suggestions of solutions to help solve problems. Another good example of an important factor is the providence of positive results when given a task and also helping each other finishing jobs allocated to them. In addition to that, optimistic people are able to adhere to all organizational ethics and rules. This is advantageous to social innovation success, as employees are able to practice better organizational behavior leading to inter-organization success.

### Resilience as an Important Antecedent for Effective Co-design of an Information System

In most organizations, resilience is an essential element that enables individuals to overcome most challenges by formulating approaches that help in problem-solving. This proposition explains how resilience is an important antecedent for an effective information system (IS). This is due to various occurrences such as specialized hackings, acts of workers, failure of equipment, and natural calamities, such as a flood, that compromise the effectiveness of the IS. As a result of these occurrences, there is a need for organizations to formulate and implement various approaches that enhance the resilience of the Information system so that they can be able to withstand the occurrences (McDaniels et al., 2008). With increased resilience, organizations will be able to achieve their goals and objectives.

Organizations are supposed to realize the need for cyber security through the formulation of important approaches that will ensure they facilitate better secure and resilient electronic systems that will maximize the outcomes of the organization. Moreover, organizations should implement the following models that will ensure improved resilience of the IT system: Preparation, Endurance, response, and recovery models.

During preparation or planning, process organizations should be able to ensure they take into consideration various aspects such as the advance in technology (Umble et al., 2003). Preparation is a model that is directly linked to the effective co-design of information systems. Some of the activities that lead to increased resilience of the IS include conduction of threat assessment programs, as well as the provision of effective incident response mechanisms and the implementation of a proper governance structure. Employees should ensure they implement effective approaches that will lead to increased resilience of the information system.

Endurance is a model that improves the IS through the implementation of effective mechanisms. It helps prevent any risk factors from taking place in the organization, leading to positive outcomes. Application of the response and recovery model helps in identification of the risks and also in the formulation of important strategies to overcome; this then leads to increased resilience in the co-design of IS.

### Self-Efficacy Is an Important Moderator for an Effective Information System

Confidence is a key factor that has led to effective information systems (Kim et al., 2016). The following factors have contributed immensely to an effective information system. Most innovative ideas are created based on people’s confidence (what they believe and trust) and the commitment they have to ensure their goals are accomplished (Bandura, 1997). Trust and commitment of workers in the IT sector have a great impact on realizing an effective information system. As a result, the trust of senior officials in their workers about key detailed information concerning the improvement of the IS will help improve the optimization of the organization’s objectives. Confidence is a part of human nature but most individuals may lack it. In most organizations, confidence is a necessity for workers and managers, since some ideas or opinions are against the social norms (Lewis and Sambamurthy, 2003).

Self-efficacy is able to facilitate a moderately effective system by being able to create a bridge by which people come together and share knowledge acquired about their organization (Agarwal et al., 2000; Thatcher and Perrewé, 2002; Fagan et al., 2003; Hasan, 2006; Torkzadeh et al., 2006; Kwon et al., 2007; Scott and Walczak, 2009). Confident people tend to be attracted to others, since they believe in each other’s capabilities. It is through this that they trust each other, being able to share with each other information attained, and thus benefitting the avenue through which knowledge is passed in an organization.

Furthermore, most ideas are based on general thoughts and depend a lot on confidence for them to be truly actualized. Employees need to cultivate confidence in themselves to be able to generate ideas and believe that all will work out in the long run. This leads to an effective IS, since workers are able to suggest their opinions, some of which are beneficial to the information system.

Confidence is also positively related to the employee’s performance in the workplace (Stajkovic and Luthans, 1998; Lwoga and Chigona, 2019). It is a human attribute that can be gained or lost and is able to affect a person’s level of energy and sense of self-worth. Therefore, it is advisable for employees to have high self-efficacy, which leads to high self-esteem that will positively impact their level of performance and produce better outcomes in organizations leading effective IS in their organizations. Confidence is a relevant asset for workers in an organization, since people communicate effectively and seek ideas enabling them to solve problems. In addition to that, confident workers promote organizations to higher levels compared to those who are not. Therefore, employees in the IT sector should cultivate the art of confidence to enhance the effectiveness of the system through hard work.

### The Significance of Hope That Helps Information Systems Determine and Actualize Its Prospects

Hope is an important factor that helps Information systems in the actualization of their prospects. Most employees in the IT sector are obligated to work hard with a determination that IS goals will be realized. With hope, various strategies have been formulated in different organizations with a belief that all difficulties can be overcome (Ybasco et al., 1996).

Hope is essential in organizations, since employees are able to work hard with a determination that their services will be upgraded, such as better insurance medical coverage. Hard work and the hope of better services yield positive results in organizations, since the IT department will be able to accomplish their objectives. Therefore, hope is positively related to improved performance in organizations, and it is important for all employees in the IT sector in an organization to cultivate the culture of hope to increase the effectiveness of IS.

Hope is effective in organizations, since individuals with hope believe that each employee and senior official has a responsibility to ensure that the organizations achieve their goals. Therefore, with this hope, employees support and interact well with each other by respecting everyone’s opinions. Some of these opinions help solve the most critical IS technical problems, leading to a more effective IS. Additionally, IT employees are also able to enhance their security and safeguard confidential information in their systems from cybercriminals.

### Inter-Organizational Information System Is Effective in Social Innovation Success

#### Relationship Between Inter-Organizational IS and Social Innovation

As has already been discussed, social innovation entails coming up with logistics that are tailored to meet the social and environmental needs of clients (Bresciani et al., 2016). That said, there needs to be an appreciation of the fact that the modern market is aggregated, and the production of similar goods heightens competition. It follows, therefore, that most businesses will pounce on the slightest chance to gain a competitive edge over rivals. To begin the cycle of social innovative success, research is required to determine the social as well as environmental needs that need to be addressed. According to Nilashi et al. (2016), market research is a function of the marketing department of the organization, which traditionally employs the use of a salesperson.

This is where the internet and networking align in a bid to cover more market and stimulate sales of the social innovative idea so that more market can be covered (Gil-Garcia and Sayogo, 2016). The author is of the view that the internet provides a good platform for interaction with buyers in real time, not to mention in a wide geographical coverage. However, in a stiffly contested market, the effects of sales promotions conducted by small and medium enterprises (SMEs) may not have far-reaching effects. It is also against this backdrop that an additional significance of the online platform for sales promotions is manifested.

In a bid to curb the pressures from macro enterprises, SMEs may cluster to launch joint efforts in their competitive and sales promotion approaches (Gil-Garcia and Sayogo, 2016). There may be a variety of mechanisms through which such ambitious alliances can be established, but by far, inter-organizational IS proves to be among the most effective methods. What this implies is that SMEs will pool resources and ideas together in a common Information System. It follows, therefore, that trust between or among such SMEs is of utmost significance, since each trust the other to act in the best interest of the entire alliance. Inter-organizational information systems also allow the aggregated SMEs to share the market base as well as information and resources (Mignerat and Rivard, 2009). An SME that is not well established in as far as e-commerce goes can, for instance, leverage the resources of a partner with a stronger technological base (Feindt et al., 2002). This will not only shield SMEs from intense competition but also enable them to have social innovation success, since their commodities aimed to meet social and environmental needs will ultimately reach wider market coverage. In summary, the benefits of an inter-organizational IS can be described as twofold. On one side it provides an interactive platform for organizations and their customers, and on the other side, it provides an outlet for socially innovated products. Business enterprises, such as SMEs that trade in social innovations, are thus able to meet their sole business purpose (selling), which further emphasizes the success of inte-organizational IS for social innovation success.

### Effectiveness of Inter-Organizational IS for Social Innovation Success

Effective inter-organizational IS facilitate social innovation success through improving the following factors.

#### Smoothened Supply Chain

The sole objective of producing a commodity tailored to meet the social needs of consumers is sales (Crain and Abraham, 2008). That said, numerous organizations trade in the same products, and it is not always guaranteed that commodities will be sold. It follows, therefore, that efficiency in distribution and customer relations are important in establishing meaningful relationships between social innovation and sales success. One strategy that an Inter-organizational information system brings to the table through the application of the internet is electronic marketing. Electronic systems can be developed among and between organizations for connection with a large set of potential buyers (Liang, 2015). Successful in such aspect, critical informational and physical resources could be mobilized more effectively in the supply chains that supporting cross-sector collaborations for social innovation. This fosters the success of social innovation. Put differently, through supply chain relationship improvement, inter-organizational IS could increase social innovation success by level up the functions the supporting supply chain can play. As an example, in such a kind of social innovation as food security initiative, an effective inter-organizational IS could strengthen the function of the supporting food supply chains by flowing the food requirement and offer information faster and more completely, leading to a much less wasted implementation of the new (socially innovative) food security initiative.

#### Giving Larger Firms Initiator Roles in Social Innovation Campaigns

According to Liang (2015), the success of an industry can simply depend on the availability of a company that plays the lead role. Tentatively, social innovation campaigns can be initiated by larger companies with a broad technological base. Owing to the fact that most macro enterprises have smaller businesses operating under their canopy, influencing industry action and change can be easy. On the other hand, however, the leading firm need to coordinate with the government units and other smaller sized business partners well, which demand a good inter-organizational IS. With such support, the leading company can initiate the inter-organizational IS program and bring smaller businesses onboard by also enabling their less-endorsed partners. This in effect means that macro enterprises have the ability to “orchestrate” for desirable changes in social innovation by promoting desired practice through intense communication and emphasis.

#### Trust Building

The success of most partnerships is based on trust (Lim et al., 2015). Companies with vested interests in performing a task have to establish a way to trust each other. For example, Kroger the US’s largest supermarket chain has managed to attract numerous investors over the years due to its merging with Teeter in a manner that embraces mutual trust and accountability (Clare, 2013). One of the most critical necessities of inter-organizational trust building is that instant information sharing and communication are necessary tasks among collaborating partners. Through the establishment of an effective inter-organizational IS, companies are able to share information amongst themselves. Gradually, this builds trust among the partnering companies, meaning that they can act on behalf of each other.

#### Collaborative Quality Assurance Approaches

Customer satisfaction is an integral part of sustainable business practice, yet customs surrounding this very sensitive matter vary greatly from one company to another (Mignerat and Rivard, 2009). Essentially, business institutions have quality assurance departments tasked with the role of ensuring that social innovations are designed to meet the specific needs of consumers (Social and environmental needs). As has already been mentioned, quality assurance practices vary. It may be prudent if several companies, through an inter-organizational IS, share their respective quality assurance practices in an online platform. The best practices can be selected from within the proposed practices and adopted for implementation. Such collaborative quality assurance approaches may also be implemented by information and technological sharing, which is a key to success for businesses in cooperation with each other, especially in matters relating to marketing, as well as competition (Liang, 2015). Through cooperation, businesses are able to share innovation information that can be used to standardize the products being produced by partners, which means that socially innovative products will appeal uniformly to buyers. Anyone outside the fold will most certainly experience competitive pressures. Additionally, through inter-organizational IS, companies are able to share technological information easily, which further gives them a strong stance in ensuring that social innovation is a success.

## Conclusion and Implications

The changing demographics of the business world today make it a prerequisite that organizations stay vigilant not only about market dynamics but also about market needs. It is important that businesses identify the social innovations that need to be developed to foster customer satisfaction, while at the same time giving them a competitive edge against rivals. This is where organizations must appreciate the value of the two factors of business cooperation and technology. In effect, the development of an inter-organizational information system should be emphasized. In so doing, it must be ensured that the psychological state of all the members of MIS development team is positive to enable the team to willingly execute the objectives of the business. Long term benefits, such as improved supply chain and trust building, will go a long way in ensuring that businesses meet their objectives. There are currently prolific studies that are relevant to PsyCap, including the first meta-analytical review (Avey et al., 2008), and a review of the psychometric properties of the PsyCap questionnaire (PCQ) (Dawkins et al., 2013). Although this work provides an important first step, proving the accuracy of the concept and establishing some of the most effective boundary conditions for PsyCap represent unresolved problems that were identified through our systematic review of the prior studies (Newman et al., 2014).

There are companies that have a wider retail base than others in diverse industries. What this implies is that companies will explore merging options as a subsidiary move to foster cooperation. Merging companies will seemingly be a in good position to share resources such as information and technology more transparently. The growth of SMEs will, in such cases, be inevitable because of the perceived benefits of large capital outlays as well as resource access. The only downfall that needs to be critically evaluated is the effect that large companies playing these roles will bring. The bigger companies will tailor the entire inter-organizational IS, for instance, to suit their own operational philosophies. An SME’s operations are likely to play a support role, meaning that some of their objectives will not be met. In the long run, this could lead to acquisitions, and hence permanent loss of smaller organizations.

This research contributes by offering inspiration for future studies to link the micro- and macro-level aspects of social innovation and entrepreneurship phenomena. The micro-level studies of the phenomena traditionally focus on the individual foundations that explain the formation of decisions, behaviors, or changes in the social innovation/entrepreneurship activities (Liao and Welsch, 2005; Zampetakis and Moustakis, 2006; Wu and Wu, 2008; Ferreira et al., 2012; Moghavvemi and Akma Mohd salleh, 2014). However, a context-specific discussion for those individual factors is lacking, thus making the generalizability impact of those studies lower. On the other hand, traditional macro-level studies for social innovation/entrepreneurship focus on overall patterns of the collectives/stakeholders for such activities (Gumusluoglu and Ilsev, 2009; Ruvio and Shoham, 2011; Ozkaya et al., 2013; Glaser et al., 2015; Kobarg et al., 2017), weakening the rationalization of why those patterns are emergent. We hope our study will encourage future research to transcend traditional boundaries and enrich research possibilities.

This study furthers the body of psychological capital on organizational commitment and citizen behavior; however, some research limitations and future works of this study may be considered in the near future. First, the possible antecedents, consequents, mediators and moderators of psychological capital and organizational citizen behavior may exist in different research areas and contexts (Hu et al., 2018; Bockorny and Youssef-Morgan, 2019; Carmona-Halty et al., 2019). The future work should make a deeper search and comparison from the current research results. Second, this study mainly focused on the contribution to propose a conceptual model and research propositions from the discussion and exploration of existing and classical literature. However, the further study could modify and improve the proposed model of this study via empirical data.

## Author Contributions

YT conceived and designed the research, wrote, and revised the manuscript. Y-FS provided guidance throughout the whole research process.

## Conflict of Interest Statement

The authors declare that the research was conducted in the absence of any commercial or financial relationships that could be construed as a potential conflict of interest.
